# The Tryptophan Decarboxylase in *Solanum lycopersicum*

**DOI:** 10.3390/molecules23050998

**Published:** 2018-04-24

**Authors:** Xin Pang, Yanping Wei, Yuan Cheng, Luzhao Pan, Qingjing Ye, Rongqing Wang, Meiying Ruan, Guozhi Zhou, Zhuping Yao, Zhimiao Li, Yuejian Yang, Weicheng Liu, Hongjian Wan

**Affiliations:** 1 Suzhou Polytechnic Institute of Agriculture, Suzhou 215008, China; pxtracy916@163.com; 2 Institute of Vegetables, Zhejiang Academy of Agricultural Sciences, Hangzhou 310021, China; weiyanping0415@163.com (Y.W.); chengyuan1005@126.com (Y.C.); jingqingye2013@126.com (Q.Y.); rongqingw2012@gmail.com (R.W.); ruanmy@163.com (M.R.); chinazhougz@163.com (G.Z.); zjyzp@163.com (Z.Y.); zhimiaoli@mail.zaas.ac.cn (Z.L.); younghz@163.com (Y.Y.); 3 College of Horticulture and Gardening, Yangtze University, Jingzhou 434023, China; Lulu_1679@163.com; 4 Zhejiang Key Laboratory of Exploitation and Preservation of Coastal Bio-resource, Zhejiang Mariculture Research Institute, Wenzhou 325005, China; lwch80@126.com

**Keywords:** tryptophan decarboxylase, structural features, expression profiles, phylogenetic relationship

## Abstract

Melatonin plays an important role in plant growth, development, and environmental stress. In this study, a systematic analysis of tomato tryptophan decarboxylase (SlTrpDC), which is the first enzyme of melatonin biosynthesis, was conducted by integrating structural features, phylogenetic relationships, an exon/intron feature, and a divergent expression profile. The results determined that the tomato genome encoded five members (*SlTrpDC1*-*SlTrpDC5*). The phylogenetic relationships indicated that gene expansion was proposed as the major mode of evolution of the *TrpDC* genes from the different plant algae species to the higher plants species. The analyses of the exon/intron configurations revealed that the intron loss events occurred during the structural evolution of the TrpDCs in plants. Additionally, the RNA-seq and qRT-PCR analysis revealed that the expression of the *SlTrpDC3* was high in all of the tested tissues, while the *SlTrpDC4* and *SlTrpDC5* were not expressed. The expression patterns of the remaining two (*SlTrpDC1* and *SlTrpDC2*) were tissue-specific, which indicated that these genes may play important roles within the different tissues. No expression difference was observed in the tomato plants in response to the biotic stresses. This study will expand the current knowledge of the roles of the *TrpDC* genes in tomato growth and development.

## 1. Introduction

Melatonin (*N*-acetyl-5-methoxytryptamine) is identified as an indoleamine with an unstable form that exists widely in various tissues of higher plants, such as the root, stem, leaf, flower, fruit and seed tissues [1,2,3]. The first research reports in 1995 found that melatonin exists in almost every plant species [4,5]. Moreover, these studies have shown that melatonin plays an important role in the circadian cycle regulation [6,7,8], broad-spectrum antioxidant [9,10,11,12], biotic and abiotic stresses [13,14,15,16], seed dormancy, and in plant growth and development [17,18,19,20,21]. Meanwhile, plants which were treated with specific concentrations of exogenous melatonin could appropriately regulate their resistance mechanisms and metabolic processes during the growth and development phases [8,22,23,24,25]. 

In recent years, research studies have found that the melatonin synthesis in plants involved four key enzymes, which included TrpDC [22,26]. However, when compared with that of animals, the plant serotonin was first purportedly synthesized by the catalysis of tryptophan decarboxylase (TrpDC), which is followed by tryptamine 5-hydroxylase (T5H), rather than the tryptophan 5-hydroxylase (Trp5H), and aromatic l-amino acid decarboxylase (AADC) in animals [27,28]. The studies reported that TrpDC with a higher expression level and the enzyme encoded by TrpDC with a higher activity contributed to the accumulation of tryptamine in transgenic tobacco [29]. Kang et al. reported that, when compared to the wild-type plants, the serotonin biosynthesis was directly related to TrpDC, and that by using a transgenic method in rice, it was detected to be 25-fold and 11-fold higher in the leaves and seeds, respectively [30]. Additionally, the research results also determined that the catalytic reaction of the TrpDC was considered a rate-limiting step in the melatonin biosynthetic pathway, which was based on the experimental results of the enzymatic activity [27,31]. Therefore, the accumulated evidence has demonstrated that TrpDC is a very important enzyme for the biosynthesis of melatonin.

Tomato, as a model plant, has become an excellent material for research studies with regard to interpreting the various life activities of plants. Previously, according to enzyme-linked immunosorbent assays, studies conducted by Okazaki et al. showed that melatonin levels in the roots, stems, leaves, flowers, fruits, seedlings and seeds of tomato plants ranging from 1.5 to 66.6 ng/g fresh weight could be detected [32]. Sun et al. recorded that exogenous melatonin treatment significantly promoted the ripening, and improved the tomato fruit quality during the post-harvest life [33]. Arnao and Hernández-Ruiz determined that the melatonin in tomato plants undergoing variable conditions had a higher melatonin content [34]. In addition, the researchers reported that melatonin not only induces drought and heat tolerance [35,36] but also cadmium stress in tomato plants [37,38,39]. Overall, melatonin was found to play a very important role in regulating the growth and development, as well as controlling the environment adaptation of tomato plants. In this study, a comprehensive analysis of the TrpDCs in tomato plants was performed by the integration of structural features, phylogenetic relationships, and expression profiles of the tomato plants’ various tissues. This study will not only contribute to the understanding of the evolutionary patterns of the *TrpDC* genes in plants, but also lay a foundation to decipher the important function of *SlTrpDCs* in regulating the melatonin biosynthesis in tomatoes.

## 2. Results

### 2.1. The Tomato Genome Encoded Five SlTrpDC Genes 

By using the amino acid sequence of the pyridoxal-dependent decarboxylase conserved domain as a query, a BlastP tool was applied to the tomato genome database of the Sol Genomics Network (SGN, http://solgenomics.net/). A total of five candidate non-redundant *SlTrpDC* genes were then identified and designated as follows: *SlTrpDC1* (*Solyc07g054860*); *SlTrpDC2* (*Solyc07g054280*); *SlTrpDC3* (*Solyc09g064430*); *SlTrpDC4* (*Solyc03g044120*); and *SlTrpDC5* (*Solyc03g045020*). The gene name, ID, and location, as well as the number of exon, protein size, molecular weight (MW), and isoelectric point (p*I*) of the *SlTrpCDs* are shown in Table 1.

### 2.2. Sequence Analysis and Homology Modeling of the SlTrpDC Proteins 

**Figure 1:**
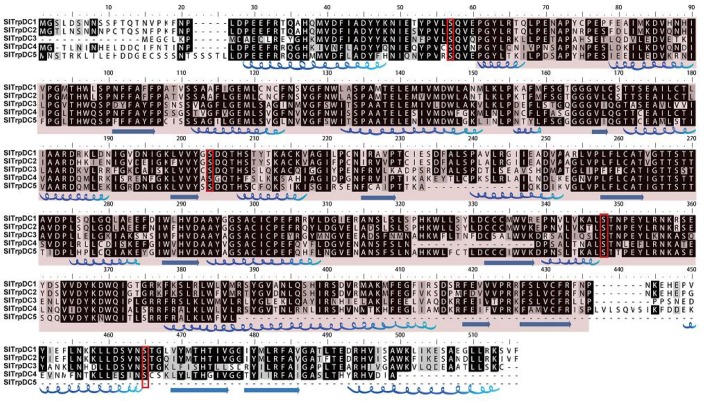
The characterization of the sequence and secondary structure of the SlTrpDC proteins. Multiple sequence alignments were performed using Clustal. The secondary structures were predicted by using a PSIPRED tool. The α-helices and β-loops are denoted with blue. The kinase-specific phosphorylation site was predicted by KinasePhos 2.0 and marked with a red box.

In order to further explore the structural features, a detailed sequence alignment and a prediction of the secondary structures of the SlTrpDC proteins were performed, and these are shown in Figure 1. It was determined that the functional domain of the *SlTrpDC* genes, the pyridoxal-dependent decarboxylase domain, was conserved. The four serine phosphorylation sites (labeled with a red box) were highly conserved, with the exception that the partial sequence, the carboxy-terminal of *SlTrpDC5*, was lost. The prediction regarding the secondary structures showed that the *SlTrpDC4* contained shorter β-loops, and the *SlTrpDC5* contained not only shorter β-loops but also deficiencies of the four α-helices and two β-loops (Figure 1A). The comparative analysis determined that the *SlTrpDC1* and *SlTrpDC2* had a high sequence identity (90.8%), while the lowest sequence identity (56.2%) was observed between the *SlTrpDC1* and *SlTrpDC3* (Table 2). 

### 2.3. Phylogenetic Relationships and Structural Characteristics 

**Figure 2:**
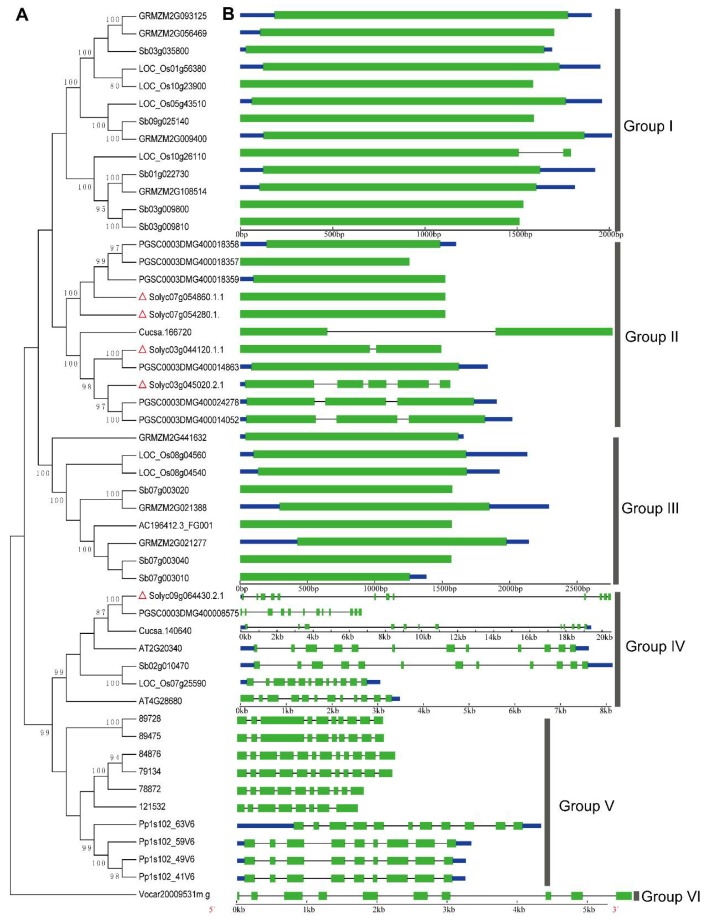
Phylogenetic relationship and exon/-intron structure of the *SlTrpDC* genes. (**A**) The neighbor-joining phylogenetic tree was inferred from the amino acid sequences alignment of the *TrpDC* genes. (**B**) The exon-intron structures are the filled green boxes (exons) and lines (introns). The blue box showed untranslated region. Red triangles showed *TrpDC* genes from *Solanum lycopersicum*. Bootstrapping (1000 replicates) was used to evaluate the degree of support for a particular grouping pattern in the phylogenetic tree. Branch lengths were assigned by pairwise calculations of the genetic distances, and missing data were treated by pairwise deletions of the gaps. The orthologous TrpDC genes involved in the phylogenetic tree include the dicots (*S. lycopersicum*: *Solyc07g054860.1.1*, *Solyc07 g054280.1.1*, *Solyc09g064430.2.1*, *Solyc03g044120.1.1*, *Solyc03g045020.2.1*; *S. tuberosum*: *PGSC0003DM G400018359*, *PGSC0003DMG400018358*, *PGSC0003DMG400018357*, *PGSC0003DMG400014863*, *PGSC 0003DMG400024278*, *PGSC0003DMG400008575*, *PGSC0003DMG400014052*; *A. thaliana*: *AT2G20340*, *AT4G28680*; *C. sativus*: *Cucsa.166720*, *Cucsa.140640*), monocot (*sorghum bicolor*: *Sb02g010470*, *Sb07g003020*, *Sb07g003040*, *Sb03g035800*, *Sb01g022730*, *Sb03g009800*, *Sb03g009810*, *Sb09g025140*, *Sb07g003010*; *Z. mays*: *GRMZM2G021277*, *AC196412.3_FG001*, *GRMZM2G021388*, *GRMZM2G441632*, *GRMZM2G093125*, *GRMZM2G056469*, *GRMZM2G108514*, *GRMZM2G009400*; *O. sativa*: *LOC_Os08g 04560*, *LOC_Os08g04540*, *LOC_Os10g26110*, *LOC_Os01g56380*, *LOC_Os07g25590*, *LOC_Os10g23900*, *LOC_Os05g43510*), Pteridophyta and Bryophyta (*S. moellendorffii*: *84876*, *79134*, *89728*, *89475*, *78872*, *121532*; *Physcomitrella patens*: *Pp1s102_63V6*, *Pp1s102_49V6*, *Pp1s102_59V6*, *Pp1s102_41V6*) and algae (*Volvox carteri*: *Vocar20009531m.g*).

In order to explore the phylogenetic relationship between the TrpDC paralogues and orthologues in the plant kingdom, a neighbor-joining phylogenetic tree with 51 *TrpDC* genes from 10 different plant species was constructed using the MEGA5 program (Figure 2A). 

According to the phylogenetic tree topology, the phylogenetic tree could be divided into six groups (Groups I to VI). The TrpDCs in Group I and Group III were from monocots, and all members of Group II were from dicots. For Group IV, all members were from both monocots and dicots. Additionally, the TrpDC homologues in *Selaginella moellendorffii* and *Physcomitrella patens* were grouped into Group V, and shared a common ancestor. Then, one member from *Volvox carteri*, *Vocar20009531m.g*, was independently grouped into Group VI, which suggested that the TrpDCs originated before the divergence of green algae and land plant species, and the gene expansion events had occurred during the course of the plants’ evolution. In *Selaginella moellendorffii* and *Physcomitrella patens*, multiple *TrpDC* genes and multiple introns in each of *TrpDC* genes were observed. This is consistent with that from higher plant species.

The structural diversity of gene family members is also a mechanism for the evolution of multiple gene families, and intron loss or gain can be an important step in generating structural diversity and complexity [40]. A comparison of the exon/intron structures of *TrpDC* genes obtained from the above plant lineages was used to examine the possible mechanisms of the structural evolution of the TrpDC homologues. The images of the exon/intron structures were obtained by using an online Gene Structure Display Server (GSDS: http://gsds.cbi.pku.edu.cn), with both coding sequences (CDS) and genomic sequences. Figure 2B provides a detailed illustration of the intron and exon configurations within each of the TrpDC homologues. The results show the various numbers of introns which were found in all the genes of the TrpDC family, across the different lineage species. Within algae and low land plant species, multiple numbers of introns (8 to 10) were observed, while in high land plant species, no intron (Group I and III) or one to four introns (Group II) were found. These results indicated that the intron loss events had occurred in the higher plant species. 

### 2.4. Differential Expression Profiles of SlTrpDC Genes Based on RNA-seq and qRT-PCR

**Figure 3:**
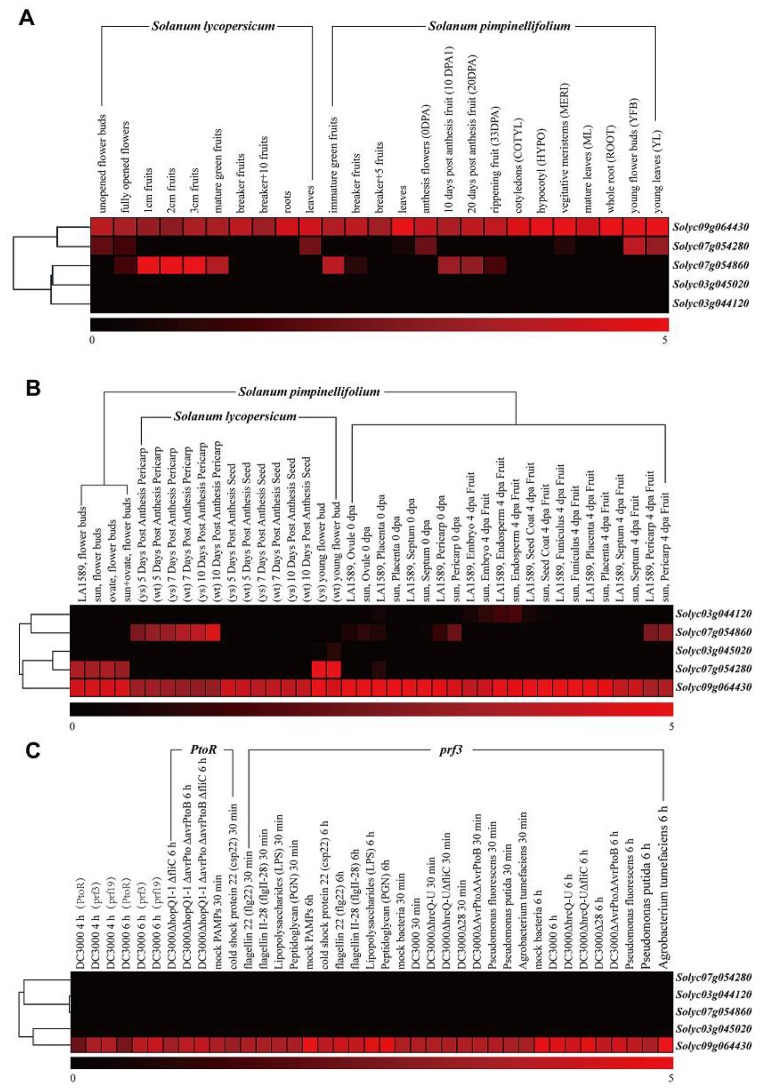
Expression profiles of *SlTrpDC* genes based on RNA-Seq in different tomato species. (**A**) the cultivated tomato (*Solanum lycopersicum*) and wild tomato (*S. pimpinellifolium*); (**B**) different tissues and organs from cultivated tomato and wild tomato (*S. pimpinellifolium*); (**C**) under biotic stresses. All RNA-Seq datasets were from Tomato Functional Genomics Datebase (http://ted.bti.cornell.edu/) and a detailed description of the samples is available in the Tomato Functional Genomics Datebase. Then, log2-transformed RPKM values were used to obtain a heatmap using the MultiExperiment Viewer software [38]. Blocks with colors indicate low (black) or high (red) transcript accumulation relative to the respective control.

The RNA-seq is a recently developed approach to transcriptome profiling which has allowed many advances in regards to the characterization and quantification of transcriptomes [41]. In order to decipher the expression pattern of the *SlTrpDC* genes among various tomato tissues, all available RNA-Seq data from the Tomato Functional Genomics Database (http://ted.bti.cornell.edu/) were downloaded. The normalized gene expression values were estimated by reads per kilo, based on per million reads mapped (RPKM). Subsequently, the log2-transformed RPKM values were used to draw heat maps using Mev4.9 software [42], and the results are shown in Figure 3. In this study, in silico expression analysis was performed on various tissues of *S. lycopersicum*. As shown in Figure 3A, the results revealed that the transcripts of the *SlTrpDC3* (*Solyc09g064430*) appeared in almost all of the various tissues of cultivated tomato, *S. lycopersicum* and the wild relative, *S. pinpinellifolium*, while the *SlTrpDC4* (*Solyc03g044120*) and *SlTrpDC5* (*Solyc03g045020*) were not detected. The expressions of the remaining two genes, *SlTrpDC1* (*Solyc07g054860*) and *SLTrpDC2* (*Solyc07g054280*), were observed in several tissues. The former was observed in 1 cm fruits, 2 cm fruits, 3 cm fruit, mature green fruits from *S. lycopersicum* and immature green fruits, 10-, 20 days post anthesis fruits and ripening fruits from *S. pimpinellifolium*, while the latter was in found in leaves from *S. lycopersicum* and anthesis flowers, young flower buds and young leaves from *S. pimpinellifolium*.

In order to further expand our knowledge of the expression profiles of the *SlTrpDC* genes in different tissues, the expression patterns of *SlTrpDC* genes were analyzed in the different tissues of the cultivated tomato, *S. lycopersicum* and the wild relative, *S. pinpinellifolium* (Figure 3B). The results showed that the transcript levels of the *SlTrpDC3* (*Solyc09g064430*) display broader expression patterns, while other members of the *SlTrpDCs* family were only expressed in specific tissues. The *SlTrpDC1* (*Solyc07g054860*) was detected in the 5–10 days post anthesis pericarp, while *SlTrpDC2* (*Solyc07g054280*) was only detected in the flowers. The expression differences of these two genes were observed between the *S. lycopersicum* and *S. pinpinellifolium.* Furthermore, the remaining two genes, *SlTrpDC4* (*Solyc03g044120*) and *SlTrpDC5* (*Solyc03g045020*), were not detected in any of the tissues. Subsequently, expression patterns of the *SlTrpDCs* in response to biotic stress treatments were conducted. The results showed no obvious difference in the expressions of the *SlTrpDCs* in response to the three various biotic factors, which included *Pst DC3000*, different bacteria, and PAMPs (Figure 3C). 

**Figure 4:**
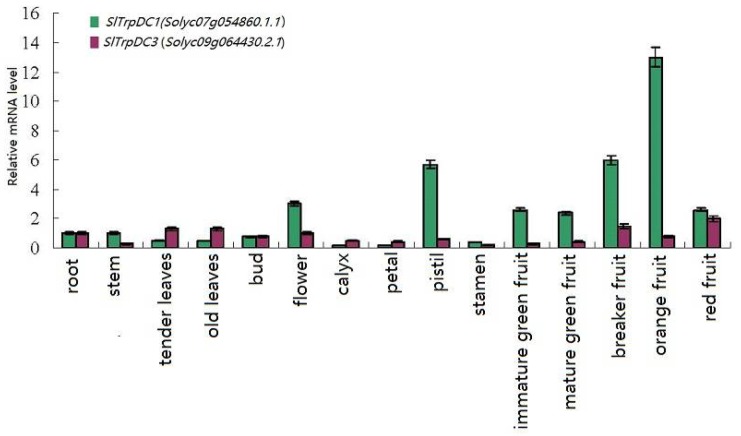
Expression profiles of the *SlTrpDC* genes in different tissues in tomato. The expression levels of these *SlTrpDC* genes in fifteen tissues were tested using RT-qPCR, including root, stem, tender leaves, old leaves, bud, flower, calyx, petal, pistil, stamen, immature green fruit, mature green fruit, breaker fruit, orange fruit and red fruit. Error bars represent standard deviations from three independent technical replicates.

To confirm the results obtained by the RNA-Seq, and in order to attempt to quantify the expression levels, a qRT-PCR was performed, and the results were compared. In this study, the expressions of the five *SlTrpDC* genes were analyzed in 15 different tissue samples, including roots, stems, tender leaves, old leaves, buds, full flowers, calyx, petals, pistil stamen, immature green fruit, mature green fruit, breaker fruit, orange fruit, and red fruit. The results showed that among these five *SlTrpDC* genes, three genes (*SlTrpDC2*, *SlTrpDC4* and *SlTrpDC5*) were not detected in any of the tissue samples. The remaining two genes, *SlTrpDC1* and *SlTrpDC3,* were expressed in all of the tissue samples, as shown in Figure 4. However, the expression levels of *SlTrpDC1* and *SlTrpDC3* were clearly different. High expression levels of *SlTrpDC1* were observed in the flower, pistil and fruit. In addition, the *SlTrpDC2* was expressed in the flower by an RNA-seq method. However, in this study, it was not detected in any of the tissues using qRT-PCR. Overall, these results were consistent with the expression of the *SlTrpDC* genes using the RNA-seq database.

## 3. Discussion

Tryptophan decarboxylase (TrpDC; EC 4.1.1.28) is a cytosolic enzyme which has been isolated from *Ophiorrhiza pumila* [43], and *Oryza sativa* [30]. Recently, TrpDC has been functionally characterized as being involved in both indole alkaloid and serotonin biosynthesis [26,44]. However, multiple research studies have shown that TrpDC may have an overwhelming advantage in the melatonin biosynthetic process, rather than the biosynthesis of IAA. For example, the accumulation of high levels of trypamine in transgenic tobacco plants with overexpressions of the *TrpDC* gene *C. roseus* did not affect the IAA levels [44]. Transgenic rice plants with over-expression of the *TrpDC* gene showed higher serotonin in their leaves and seeds compared with wild-type plants, while serotonin has been considered to be a precursor of melatonin in both plants and animals [30]. Additionally, other research study results have revealed that the *PaTrpDC* expression in tested sweet cherry samples was directly related to the melatonin production [45]. Therefore, it can be concluded that TrpDC is an important enzyme of the melatonin biosynthetic pathway.

In the current study, the identification and characterization of the *TrpDC* gene family of tomato plants is reported. Five members in the *SlTrpDCs* family were obtained from the whole tomato genome. Further analysis determined that different land plants contained similar numbers of *TrpDC* genes, which suggested that a small gene family existed throughout the high plant kingdom. The phylogenetic relationship of the *TrpDCs* from the tested plant species (from algae to higher plants) grouped all the *TrpDC* genes into multiple sub-families, and indicated that the *TrpDC* genes had evolved before the divergence of algae and plants. With regard to the land plant species, the members from pteridophyta (*Selaginella moellendorffii*) and bryophyte (*Physcomitrella patens*) were grouped into Group V, while multiple sub-families occurred in the angiosperm, including Dicotyledoneae and Monocotyledoneae. This suggested that the *TrpDC* genes originated before the divergence of the green algae and the land plant species. The algae, *Volvox carteri*, only had one member, while there were multiple members identified in the plants (Figure 2), which suggested that significant gene expansion events had occurred after the divergence of the algae and higher plants. The analysis of the intron-exon structure showed significant differences in the numbers of the introns observed in the tested plant species. In the algae and low plant species (Groups V and VI), multiple introns were identified, while in the high plant species, no introns or few introns were observed within Groups I, II, and III. These results indicated that, after *TrpDC* gene expansion in high plants, intron loss in some *TrpDC* genes had occurred during the course of the plants’ evolution. Overall, the aforementioned phylogenetic analysis, along with the exon/intron structure comparison, revealed that the gene expansion and intron loss events were the major modes of evolution of the *TrpDC* genes in the plant species. 

To investigate the possible functional differences of the *SlTrpDC* genes, further analysis of the expression patterns of the *SlTrpDC* genes, based on the RNA-Seq and qRT-PCR technology, were conducted. The results showed that the expression of *SlTrpDC3* (*Solyc09g064430*) was detected in all of the tested tissues, which suggested an important role in the growth and development of the tomato plants. The *SlTrpDC1* (*Solyc07g054860*) expression had a significant advantage during the development of tomato fruit, and *SlTrpDC2* (*Solyc07g054280*) was detected in the leaves of the tomato plants based on the RNA-Seq. These results indicated that the expressions of these two genes were tissue-specific, and they could potentially play a vital role in the fruit and flower development. However, the expression of *SlTrpDC4* and *SlTrpDC5* were not detected in any tissues by both the RNA-Seq and qRT-PCR. This suggested that the expression levels of these genes were too low to be detected in the tested tissues, or they were not expressed to any significant degree and might correspond to processed pseudogenes.

In summary, this study identified five members of the *SlTrpDC* gene family in tomato plants, and deciphered the evolutionary relationships of the *TrpDC* homologous genes in the plant kingdoms. Further analysis determined that two *SlTrpDC* genes, *SlTrpDC1* and *SlTrpDC2*, displayed tissue-specific expression profiles. This study will lay the foundation for deciphering the function of *TrpDC*s family members with regard to the melatonin synthesis in tomato plants.

## 4. Materials and Methods

### 4.1. Identification of the TrpDC Genes Family in Tomato 

An HMM profile of the TrpDC pyridoxal-dependent decarboxylase conserved domain (Pfam: PF00282) was downloaded from the Pfam protein families database (http://pfam.sanger. ac.uk/) to identify the *TrpDC* genes from the *Solanum lycopersicum* genome using HMMER3.0 (http://hmmer.janelia.org/). Default parameters were employed, and all non-redundant gene sequences were searched from the tomato genome data of SGN (http://solgenomics.net/). Subsequently, the molecular weights and isoelectric point of the SlTrpDCs deduced proteins were then predicted by using the online tool ExPASy (http://web.expasy.org/protparam/).

### 4.2. Sequence Features

Multiple sequence alignments of the SlTrpDCs protein sequences were performed using Clustal software (version 2.0) [46], and encoded by a BioEdit Sequence Alignment Editor (http://www.mbio.ncsu.edu/bioedit/page2.html). The kinase-specific phosphorylation site was predicted by KinasePhos2.0 (http://kinasephos2.mbc.nctu.edu.tw/index.html). The secondary structures and homology modeling were predicted by utilizing the PSIPRED (http://bioinf.cs.ucl.ac.uk/psipred/) and Swiss-Model (http://swissmodel.expasy.org/), respectively. The structure was viewed with a Swiss-Pdb Viewer program [47].

### 4.3. Structural Characteristics and Phylogenetic Relationships

In order to investigate the structural characteristics and phylogenetic relationships of the *TrpDC* gene family, the genome sequence, coding sequence (CDS), and protein sequence of the homologous genes of the *SlTrpDC*s from the different plant species were obtained by using Phytozome 9.1 (http://www.phytozome.net/search.php), with a threshold E value of 1e-10, and included *S. lycopersicum*, *S. tuberosum*, *Arabidopsis thaliana*, *Cucumis sativus*, *Sorghum bicolor*, *Zea mays*, *Oryza sativa*, *Selaginella moellendorffii*, *Physcomitrella patens,* and an alga (*Volvox carteri*). The schematic diagram of the intron-exon structure of the *TrpDC* genes was depicted by the online tool Gene Structure Display Sever (version 2.0) (http://gsds.cbi.pku.edu.cn/). Additionally, in order to elaborate the phylogenetic relationships in the *TrpDC* homologues of the plants, a phylogenetic tree was constructed by MEGA 5.0 software by using the Neighbor-Joining method [48,49]. A bootstrap analysis was performed by 1000 resampling replications, and then branch lengths were assigned through the pairwise calculations of the genetic distances. The missing data were treated by the pairwise deletions of the gaps.

### 4.4. Expression Analysis of the SlTrpDC Genes Based on RNA-seq and Quantitative Real-Time PCR

The widespread application of RNA-seq data has provided convenience for detecting the differential expression of genes [42]. In this study, in order to decipher the expression pattern of the *SlTrpDC* gene family in the various tissues of tomato plants, and in response to biotic stresses, all available transcriptome data of the *SlTrpDC* genes were therefore obtained from the Tomato Functional Genomics Database (http://ted.bti.cornell.edu/). The obtained expression data were then submitted to the Multiple Experiment Viewer (Version Mev 4.9) software program with a log_2_ transformation, for the purpose of generating a heat map [42]. The obtained data were hierarchically clustered based on a Pearson correlation distance with an average linkage. Additionally, a cluster analysis was performed on the rows of expression values.

To further verify the expression pattern of the *SlTrpDCs*, fifteen tissues samples were obtained, which included roots, stems, tender leaves, old leaves, buds, and full flowers, as well as the calyx, petal, pistil stamen, immature green fruit, mature green fruit, breaker fruit, orange fruit, and red fruit from *S. lycopersicum* L. var zhefen702. These tissue samples were grown in a controlled environment chamber at the Zhejiang Academy of Agricultural Sciences. The total RNA was extracted, and the first-strand cDNA was synthesized using an RNA simple Total RNA Kit (Tiangen Biotech, Beijing, China) and a TIANScript cDNA Synthesize Kit (Tiangen Biotech), respectively, in accordance with the manufacturer’s instructions. The gene-specific primers of the *SlTrpDCs* for the qRT-PCR are listed in Table 3. The real-time PCR reactions were carried out in a total volume of 20 μL, which contained 10 μL of SuperMix, 0.4 μL of each primer, 1 μL of template (10× diluted cDNA from samples) and 7.8 μL of sterile distilled water. The thermal conditions were as follows: 95 °C for 30 s; followed by 40 cycles at 95 °C for 5 s; 55 °C for 15 s; and 72 °C for 10 s. The relative gene expression values were calculated using the 2^−ΔΔCt^ method. The GAPDH was used as a reference gene for the expression analysis of the *SlTrpDC* genes in the tomato plants, and three independent replicates were then performed [50].

## Figures and Tables

**Table 1:** The *SlTrpCD* genes in tomato.

Gene Name	Gene ID	Location of Genes	Number of Exons	Gene Length (bp)	Protein Size (aa)	MW (kDa)	p*I*
*SlTrpDC1*	*Solyc07g054860.1.1*	Chr07:63043532-63045046	0	1515	504	56.54	6.28
*SlTrpDC2*	*Solyc07g054280.1.1*	Chr07:62627192-62628707	0	1515	504	56.76	5.72
*SlTrpDC3*	*Solyc09g064430.2.1*	Chr09:61653029-61660029	11	7579	487	54.47	5.73
*SlTrpDC4*	*Solyc03g044120.1.1*	Chr03:8136445-8137928	1	1484	476	53.23	6.83
*SlTrpDC5*	*Solyc03g045020.2.1*	Chr03:11305456-11307004	4	1514	374	41.73	5.81

**Table 2:** Identity levels of the SlTrpCD proteins.

Name	*SlTDC1*	*SlTDC2*	*SlTDC3*	*SlTDC4*	*SlTDC5*
*SlTDC1*	100%				
*SlTDC2*	98.8%				
*SlTDC3*	56.2%	57.1%			
*SlTDC4*	60.1%	60.1%	57.4%		
*SlTDC5*	60.7%	60.1%	60.4%	71.6%	100%

**Table 3:** Primers of *TrpCD* genes in tomato.

Gene	Name	Primer Sequence (5′-3′)
*Solyc07g054860.1.1*	*SlTDC1*	F: GCTGCACGTGATCGTAAACT R: GCAGCAACATCAGCTTCAAT
*Solyc07g054280.1.1*	*SlTDC2*	F: TTTCCTCTGTGCTACCGTTG R: GTGGGCTTAGGCTTAACGAG
*Solyc09g064430.2.1*	*SlTDC3*	F: GGTCAAGGAGGTGGAGTGAT R: AGAGCATAATCCCTGGATGG
*Solyc03g044120.1.1*	*SlTDC4*	F: CCCTGCTGCTACTGAACTTG R: CATTTGATCTCTAGCCGCAA
*Solyc03g045020.2.1*	*SlTDC5*	F: GGTACATGTTGATGCAGCGT R: ACCACCTGTTGGGATTCACT
